# Atypical variants of the cerebral arterial circle beyond the Lazorthes classification: a retrospective morphologic and neuroradiologic study

**DOI:** 10.1007/s00276-026-03916-9

**Published:** 2026-06-15

**Authors:** Ionut Bulbuc, Mihai Lupascu, Petru Bordei, Constantin Ionescu, Bogdan Marian Caraban

**Affiliations:** Faculty of Medicine, Ovidius University of Constanta, Constanta, Romania

**Keywords:** Arterial circle of the brain, Circle of Willis, Anatomical variation, Collateral circulation, Neuroradiology, Computed tomography angiography, Magnetic resonance angiography, Persistent trigeminal artery, Posterior communicating artery

## Abstract

**Purpose:**

To characterize atypical variants of the cerebral arterial circle that fall outside the Lazorthes classification and to interpret them in a contemporary neuroradiologic framework.

**Methods:**

This retrospective morphologic-neuroradiologic study analyzed 650 cases collected between 1 September 2023 and 1 March 2026: 40 dissections, including 10 injected with plastic material, 10 digital subtraction angiograms, 300 computed tomography angiograms, and 300 magnetic resonance angiograms. Typical configurations were separated from non-classifiable variants using the 22-variant Lazorthes system as the reference framework.

**Results:**

Of the 650 cases, 523 (80.5%) were assignable to the Lazorthes classification, whereas 127 (19.5%) showed atypical configurations. These included six-sided anterior variants, anatomical absence or imaging non-visualization of one or two arterial segments, and incomplete posterior anastomotic patterns caused by segmental hypoplasia. The most frequent atypical pattern was unilateral anatomical absence or imaging non-visualization of a posterior communicating artery (43 cases; 6.6%), followed by unilateral anatomical absence or imaging non-visualization of a P1 segment (17 cases; 2.6%) and bilateral anatomical absence or imaging non-visualization of the posterior communicating arteries (11 cases; 1.6%).

**Conclusion:**

A substantial proportion of cerebral arterial circles cannot be adequately described within the classical Lazorthes typology. These atypical variants are best interpreted as patterns of altered collateral design with potential relevance for CTA and MRA interpretation, cross-flow potential, vascular territory dependence, and cerebrovascular risk assessment.

## Introduction

The arterial circle of the brain, also known as the Circle of Willis, is the principal arterial collateral network connecting the carotid and vertebrobasilar circulations at the base of the brain. In neuroradiology, its configuration is clinically relevant because the architecture and caliber of its constituent segments influence collateral capacity, vascular territory dependence, and interpretation of cerebral CTA and MRA in ischemic disease, aneurysm assessment, and preoperative planning [5, 6, 8, 11, 15, 17].

For the purposes of this article, the reference group of so-called typical configurations is represented by the 22 variants described by Guy Lazorthes [7]. The present study does not revisit those classical types; instead, it focuses on variants that do not fit within the Lazorthes classification. In the present material, 523 of 650 cases were assigned to one of the 22 Lazorthes types, whereas 127 cases (19.5%) showed configurations outside that framework.

The classical concepts of strong and weak segments, as well as dominant and dominated arterial pillars, retain historical value as early hemodynamic interpretations of the Circle of Willis. However, in contemporary neuroradiology these notions are better reformulated in terms of segment caliber, symmetry, completeness of the communicating pathways, collateral capacity, and flow-dependent functional competence [3, 4, 6–8].

## Materials and methods

Morphologic assessment of the arteries constituting the cerebral arterial circle was performed in 650 cases analyzed between 1 September 2023 and 1 March 2026. The material included 40 anatomic dissections, of which 10 specimens were additionally injected with plastic material, 10 digital subtraction angiograms, 300 computed tomography angiograms, and 300 magnetic resonance angiograms. The anatomic dissections and plastic injections were carried out in the Anatomy Laboratory of the Faculty of Medicine, Ovidius University of Constanta. The radiologic material was obtained from the Medimar Radiology Clinic, which operates within Saint Apostle Andrew County Clinical Emergency Hospital, Constanta.

The study was designed as a retrospective descriptive morphologic and neuroradiologic investigation. Typical configurations were classified according to the 22 variants described by Guy Lazorthes, which were used as the historical morphofunctional reference framework. Configurations that could not be assigned to this typology were classified as atypical variants and subsequently grouped according to their dominant morphologic pattern: altered polygonal architecture, absence of one arterial segment, absence of two arterial segments, or incomplete anastomotic continuity. No formal exclusion criteria were predefined beyond technical and morphologic suitability of the material. In the anatomical series, damaged specimens or preparations that did not allow reliable evaluation of the cerebral arterial circle were not included. In the imaging series, examinations that could not be adequately processed for 3D reconstruction or that showed insufficient image quality for confident morphologic interpretation were excluded.

In the dissection material, vascular evaluation was performed after removal of the arachnoid membrane, without optical magnification. In the injected specimens, Technovit 7143 was used as the injection material. The three feeding arteries were catheterized and injected sequentially, one after another, in order to obtain clear delineation of the arterial segments participating in the cerebral arterial circle. The DSA studies were obtained on a monoplanar system using standard projections. In this subgroup, two-dimensional angiographic analysis was used predominantly for evaluation of the constituent arterial segments rather than for assessment of the entire cerebral arterial circle as a single unit. CTA examinations were acquired on a GE Revolution Evo 128-slice scanner with a slice thickness of 1.25 mm. Post-processing included maximum intensity projection and three-dimensional reconstructions. Iodinated contrast medium was administered at an injection rate of 5 mL/s, with bolus tracking monitored at the level of the aortic arch. MRA examinations were performed on a Signa Voyager 1.5T 16-channel system using time-of-flight magnetic resonance angiography. Morphologic assessment was based on both source images, for fine structural detail, and maximum intensity projection reconstructions for vascular overview.

Morphologic evaluation was performed by I.B. and C.I. Discrepancies in classification or segment interpretation were resolved by consensus. Interpretation focused on the presence, continuity, symmetry, and relative caliber of the A1, anterior communicating, posterior communicating, and P1 segments, as well as on the identification of persistent embryonic carotid-basilar anastomoses when present. In the anatomical material, complete non-identification of a segment was recorded as absence. In the imaging material, however, non-visualization was interpreted more cautiously and was considered compatible with aplasia or severe hypoplasia unless the morphology was unequivocal. Findings are reported as absolute counts and percentages. No inferential statistical analysis was performed because the aim of the study was descriptive classification rather than hypothesis testing. For the radiologic component of the study, all patients had signed informed consent allowing the use of anonymized imaging data for scientific and research purposes. The manuscript contains no directly identifiable personal data.

## Results

A total of 127 cases (19.5%) displayed atypical configurations beyond the Lazorthes classification. These variants included six-sided configurations replacing the expected anterior communicating pattern, agenesis of one or two constituent segments, persistent embryonic carotid-basilar communications, and incomplete posterior anastomotic arrangements. The most frequent atypical variant was unilateral absence of a posterior communicating artery (43 cases, 6.6%) (Table 1).

**Table 1:** Summary of atypical variants identified in the present series

Variant	Morphologic definition	Cases (*n*)	Frequency (%)	Neuroradiologic relevance
a	Six-sided configuration due to latero-lateral anastomosis of the anterior cerebral arteries	9	1.4	May simulate a very short AComA or proximal ACA fusion
b	Six-sided configuration with azygos anterior cerebral artery	6	0.9	Distal anterior circulation unification; relevant for aneurysm anatomy
c	Anatomical absence or imaging non-visualization of the anterior communicating artery	5	0.7	Reduced anterior cross-flow between the carotid systems
d	Anatomical absence or imaging non-visualization of one A1 segment	9	1.4	Marked anterior circulation asymmetry and unilateral inflow dependence
e	Anatomical absence or imaging non-visualization of one posterior communicating artery	43	6.6	Reduced ipsilateral carotid-posterior collateral continuity
e2	Persistent trigeminal artery replacing the missing posterior communicating pathway	2	0.3	Persistent embryonic carotid-basilar collateral pathway
f	Anatomical absence or imaging non-visualization of one P1 segment	17	2.6	Carotid-dependent supply of the ipsilateral PCA territory
f2	Persistent trigeminal artery associated with absent P1 segment	1	0.1	Compound embryologic collateral configuration
g	Bilateral anatomical absence or imaging non-visualization of the posterior communicating arteries	11	1.6	Markedly reduced anterior-to-posterior collateral recruitment
h	Bilateral anatomical absence or imaging non-visualization of the P1 segments, associated with basilar hypoplasia	3	0.4	Extreme bilateral carotid-dominant posterior circulation pattern
i	Anatomical absence or imaging non-visualization of one P1 segment associated with anatomical absence or imaging non-visualization of the contralateral posterior communicating artery	5	0.7	Highly unbalanced posterior collateral configuration
j1	Incomplete posterior anastomosis due to P1 hypoplasia	7	1.1	Nearly complete circle with functionally insufficient posterior communication
j2	Incomplete posterior anastomosis due to PComA hypoplasia	9	1.4	PComA visible yet functionally inadequate for collateral recruitment

## Detailed description of atypical variants

### Variant a

Six-sided configuration with latero-lateral anastomosis of the anterior cerebral arteries.

**Fig. 1:**
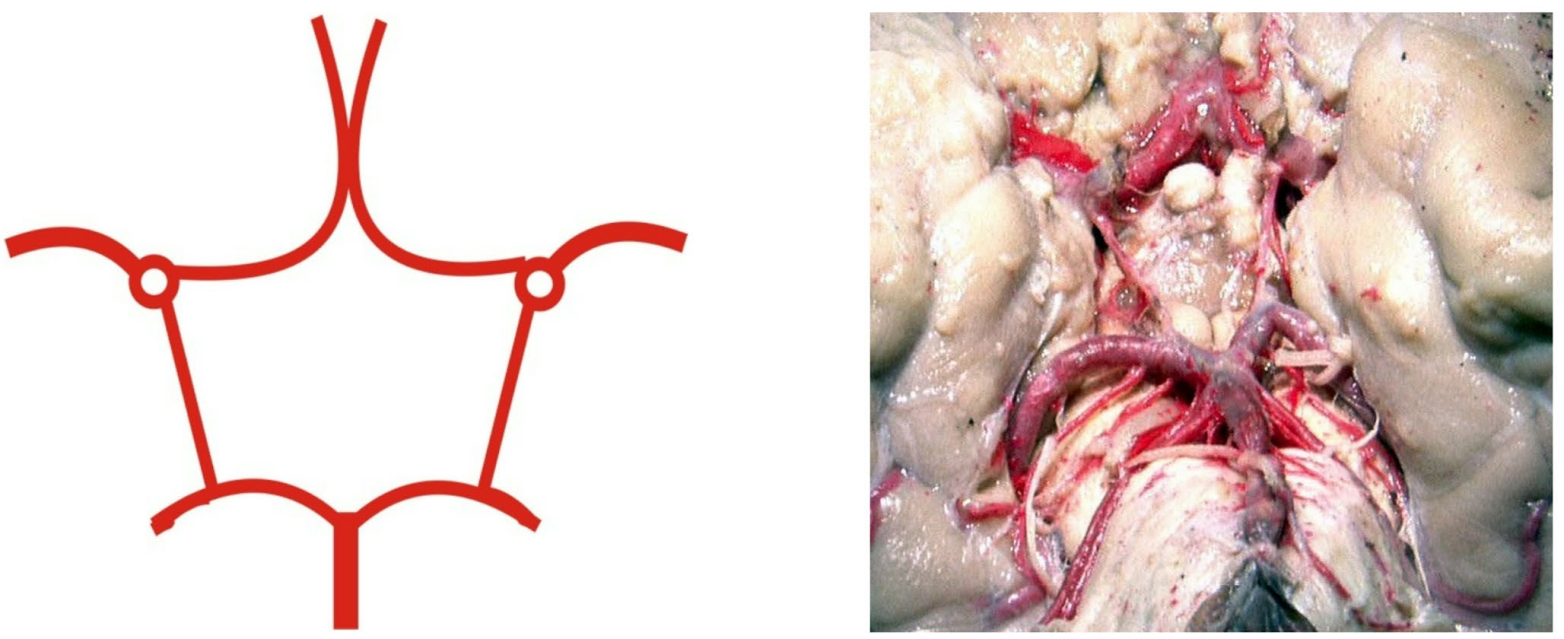
Variant a. (**A**) Schematic representation. (**B**) Representative anatomic specimen

In this pattern, the anterior communicating region is replaced by a direct latero-lateral anastomosis between the two anterior cerebral arteries, producing a six-sided arterial configuration. On imaging, this arrangement may mimic a very short anterior communicating artery or focal proximal ACA fusion. In the present series, this pattern was identified in 9 cases (1.4%) (Fig. 1).

### Variant b

Six-sided configuration with azygos anterior cerebral artery.

**Fig. 2:**
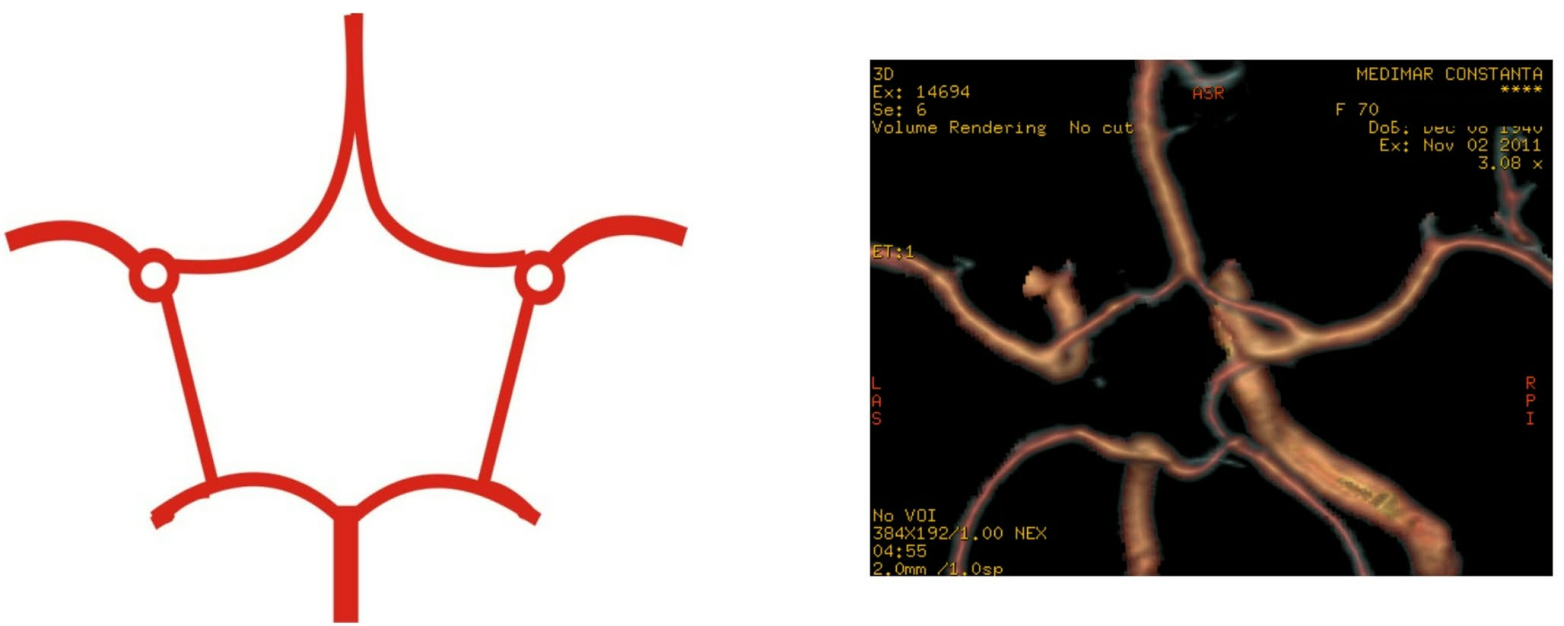
Variant b. (**A**) Schematic representation. (**B**) Representative volume-rendered CTA

This variant is characterized by fusion of the two anterior cerebral arteries at the A2 level, forming an azygos anterior cerebral artery. The finding is relevant because it modifies distal anterior circulation supply and the anatomy of the anterior midline. In the present series, this pattern was identified in 6 cases (0.9%) (Fig. 2).

### Variant c

Absence of the anterior communicating artery.

**Fig. 3:**
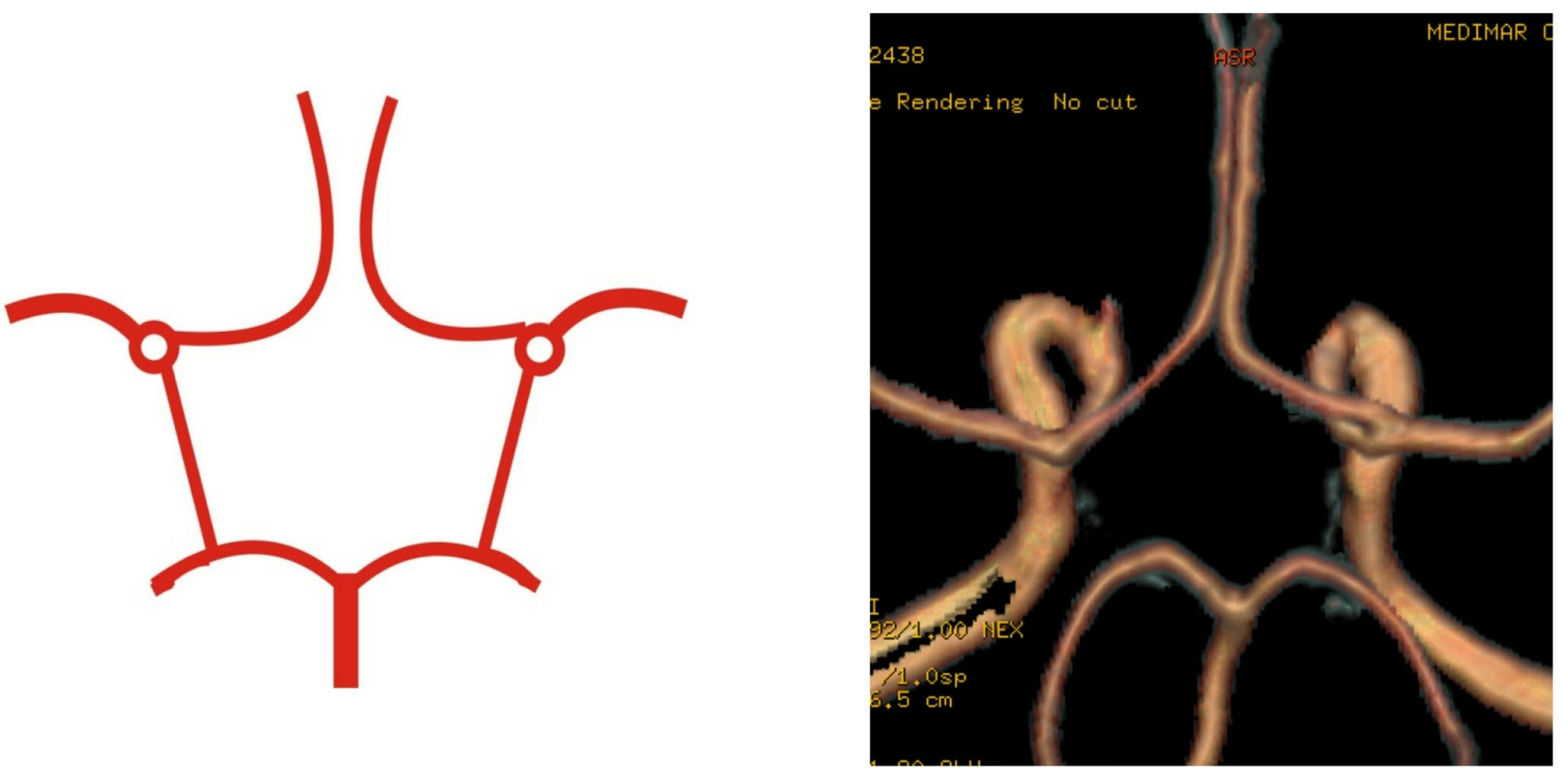
Variant c. (**A**) Schematic representation. (**B**) Representative volume-rendered CTA

This configuration represents an incomplete anterior part of the arterial circle caused by anatomical absence or imaging non-visualization of the anterior communicating artery. The main functional consequence is reduced carotid-to-carotid cross-flow potential. In the present series, this pattern was identified in 5 cases (0.7%) (Fig. 3).

### Variant d

Absence of one A1 segment.

**Fig. 4:**
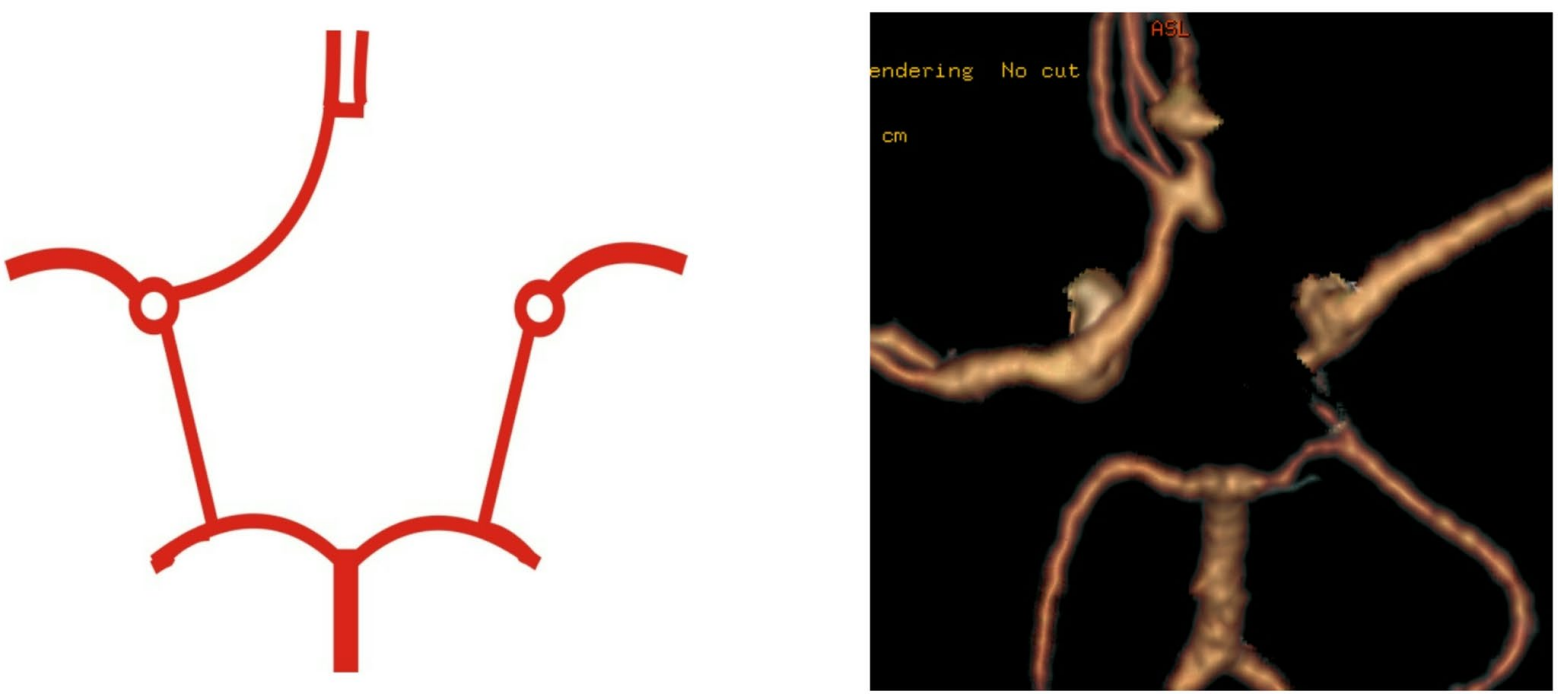
Variant d. (**A**) Schematic representation. (**B**) Representative volume-rendered CTA

This variant consists of unilateral anatomical absence or imaging non-visualization of the pre-communicating segment of the anterior cerebral artery. It creates marked anterior circulation asymmetry and causes both distal ACA territories to depend on a single carotid inflow through the anterior communicating region. In the present series, this pattern was identified in 9 cases (1.4%) (Fig. 4).

### Variant e

Absence of one posterior communicating artery.

**Fig. 5:**
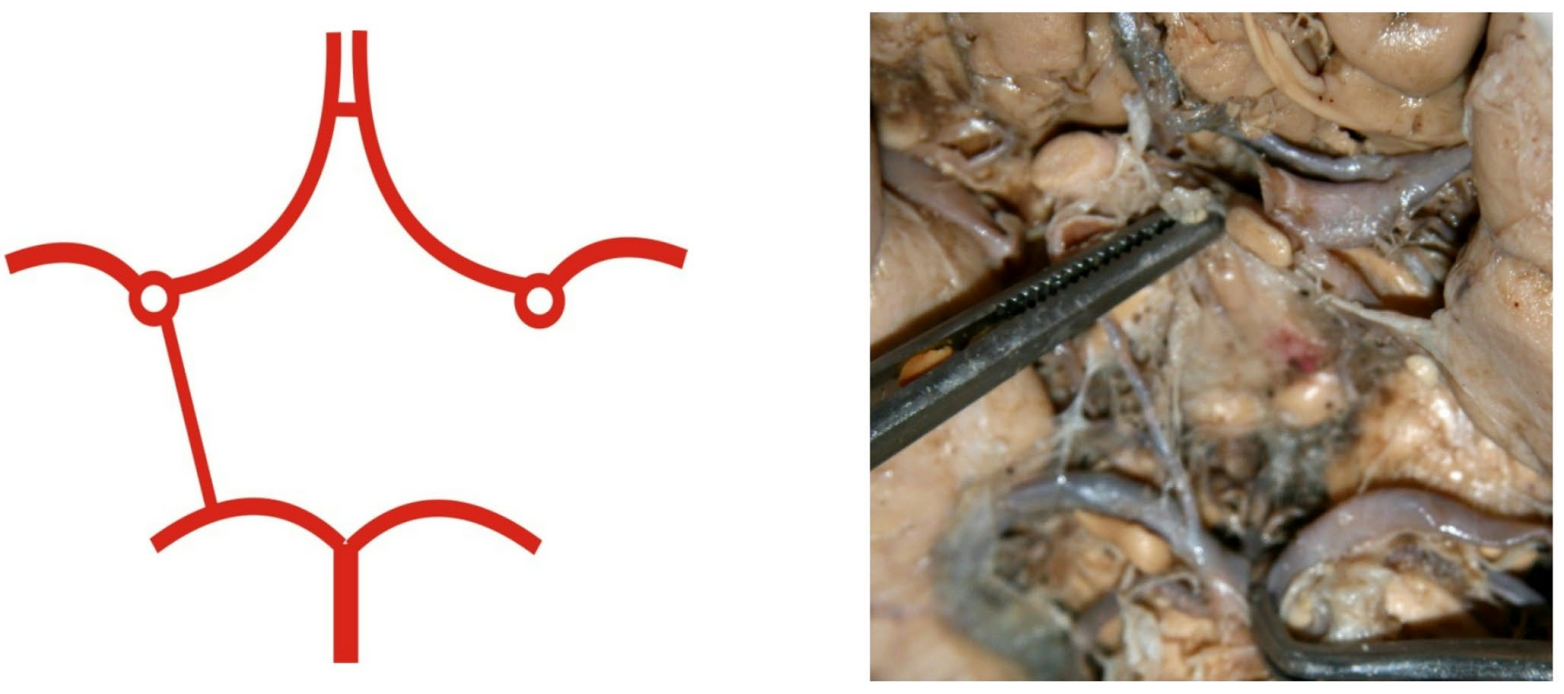
Variant e. (**A**) Schematic representation. (**B**) Representative anatomic specimen

This variant corresponds to unilateral anatomical absence or imaging non-visualization of a posterior communicating artery. It reduces carotid-posterior continuity on the affected side and may influence posterior collateral recruitment during carotid or vertebrobasilar compromise. In the present series, this pattern was identified in 43 cases (6.6%) (Fig. 5).

### Variant e2

Persistent trigeminal artery replacing the missing posterior communicating pathway.

**Fig. 6:**
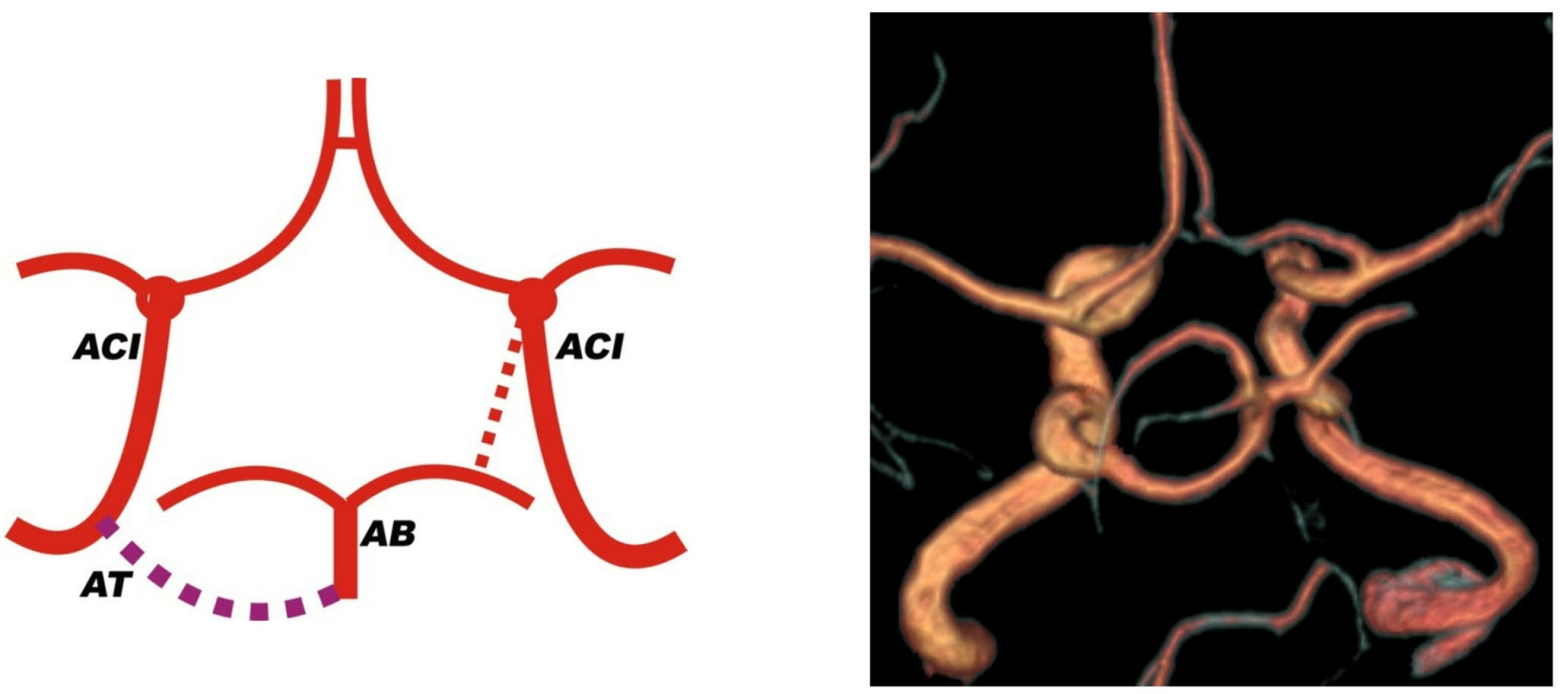
Variant e2. (**A**) Saltzman type II schematic. (**B**) Representative volume-rendered CTA showing persistent trigeminal artery

A persistent trigeminal artery may compensate for the absence of the posterior communicating artery by establishing an embryonic carotid-basilar connection. This variant preserves collateral communication, but on an atypical and developmentally distinct pathway. In the present series, this pattern was identified in 2 cases (0.3%) (Fig. 6).

### Variant f

Absence of one P1 segment.

**Fig. 7:**
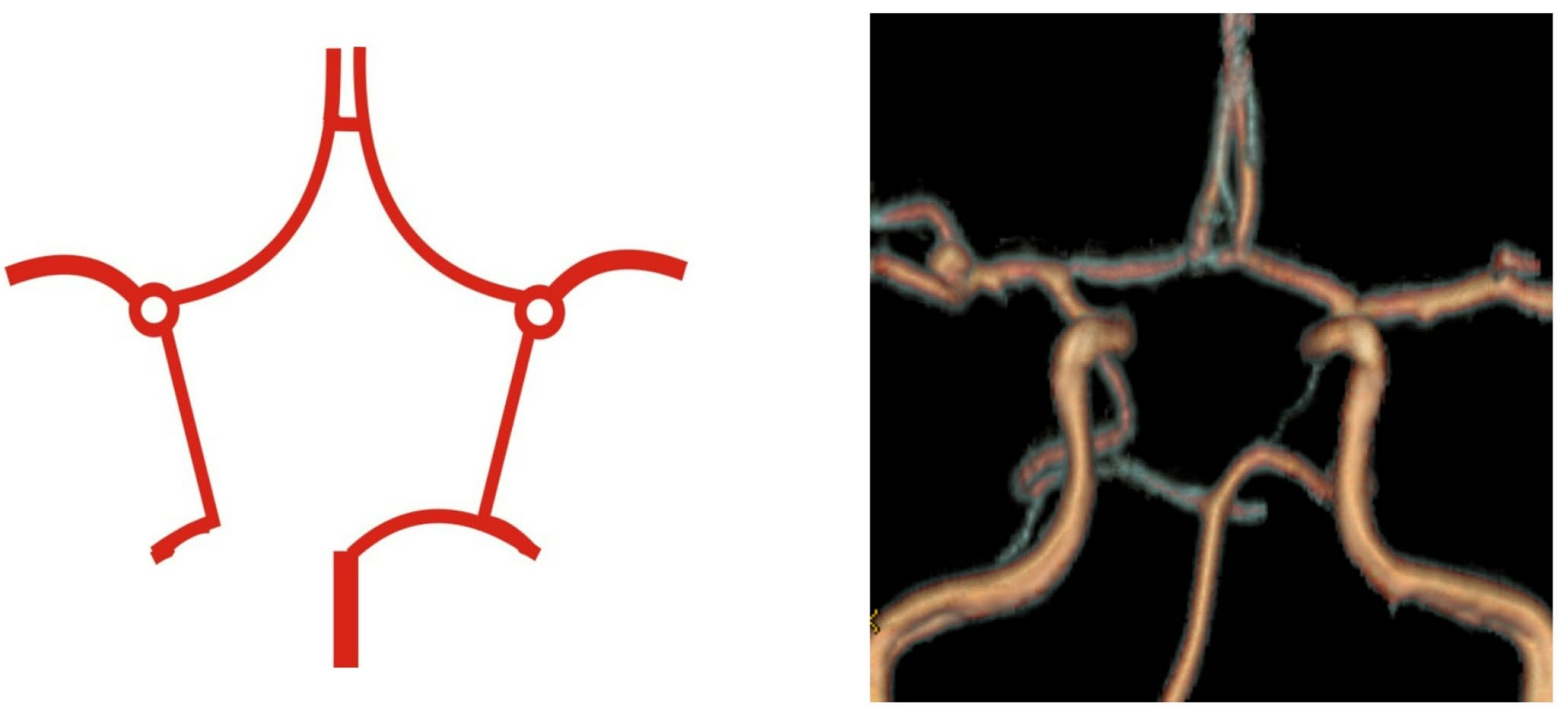
Variant f. (**A**) Schematic representation. (**B**) Representative volume-rendered CTA

This variant corresponds to unilateral anatomical absence or imaging non-visualization of a P1 segment. Functionally, it implies carotid dependence of the ipsilateral posterior cerebral artery territory and approaches a complete fetal-type posterior cerebral artery pattern. In the present series, this pattern was identified in 17 cases (2.6%) (Fig. 7).

### Variant f2

Persistent trigeminal artery associated with absent P1 segment.

**Fig. 8:**
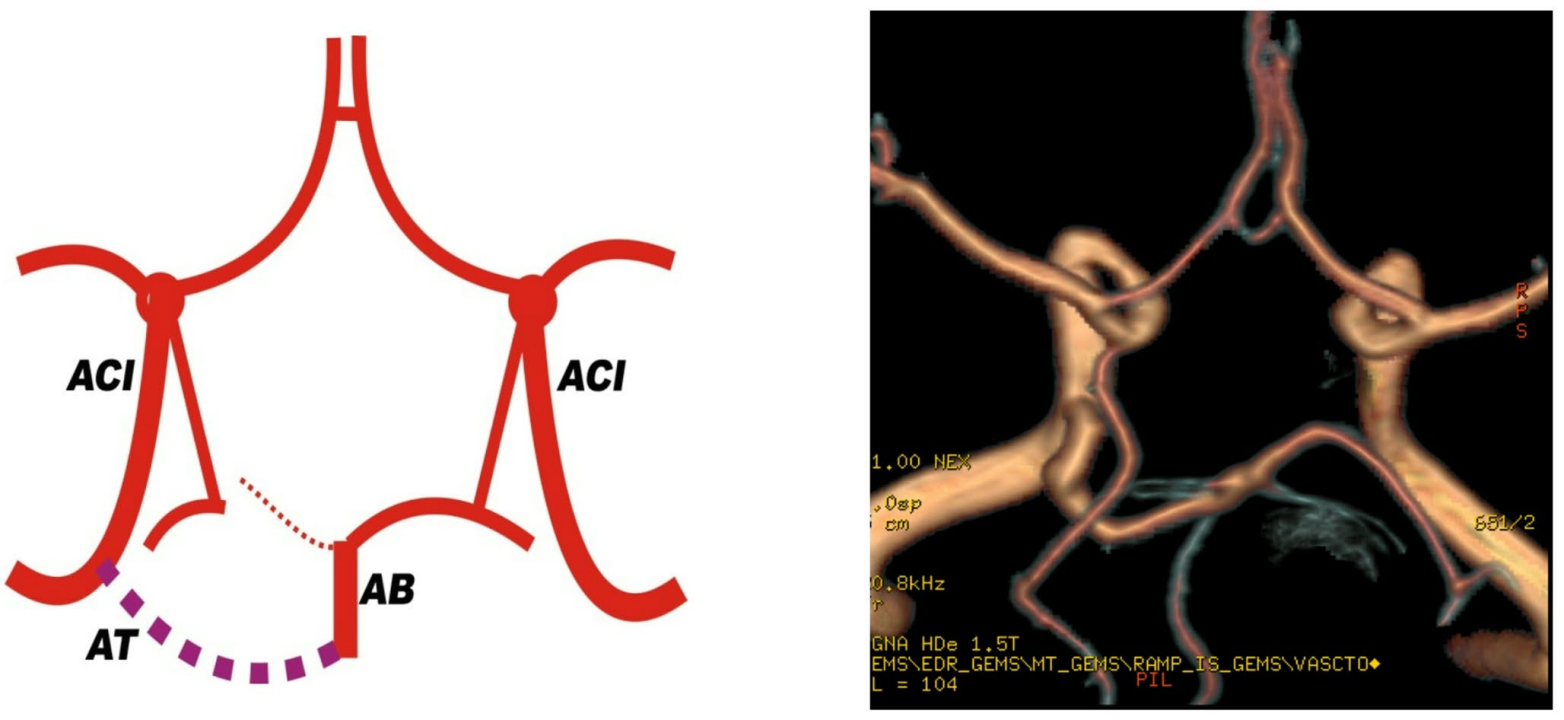
Variant f2. (**A**) Saltzman type I schematic. (**B**) Representative volume-rendered CTA showing persistent trigeminal artery with absent P1

In this compound variant, a persistent trigeminal artery coexists with absence of the ipsilateral P1 segment, preserving carotid-basilar communication while replacing the conventional proximal posterior cerebral connection. In the present series, this pattern was identified in 1 case (0.1%) (Fig. 8).

### Variant g

Bilateral absence of the posterior communicating arteries.

**Fig. 9:**
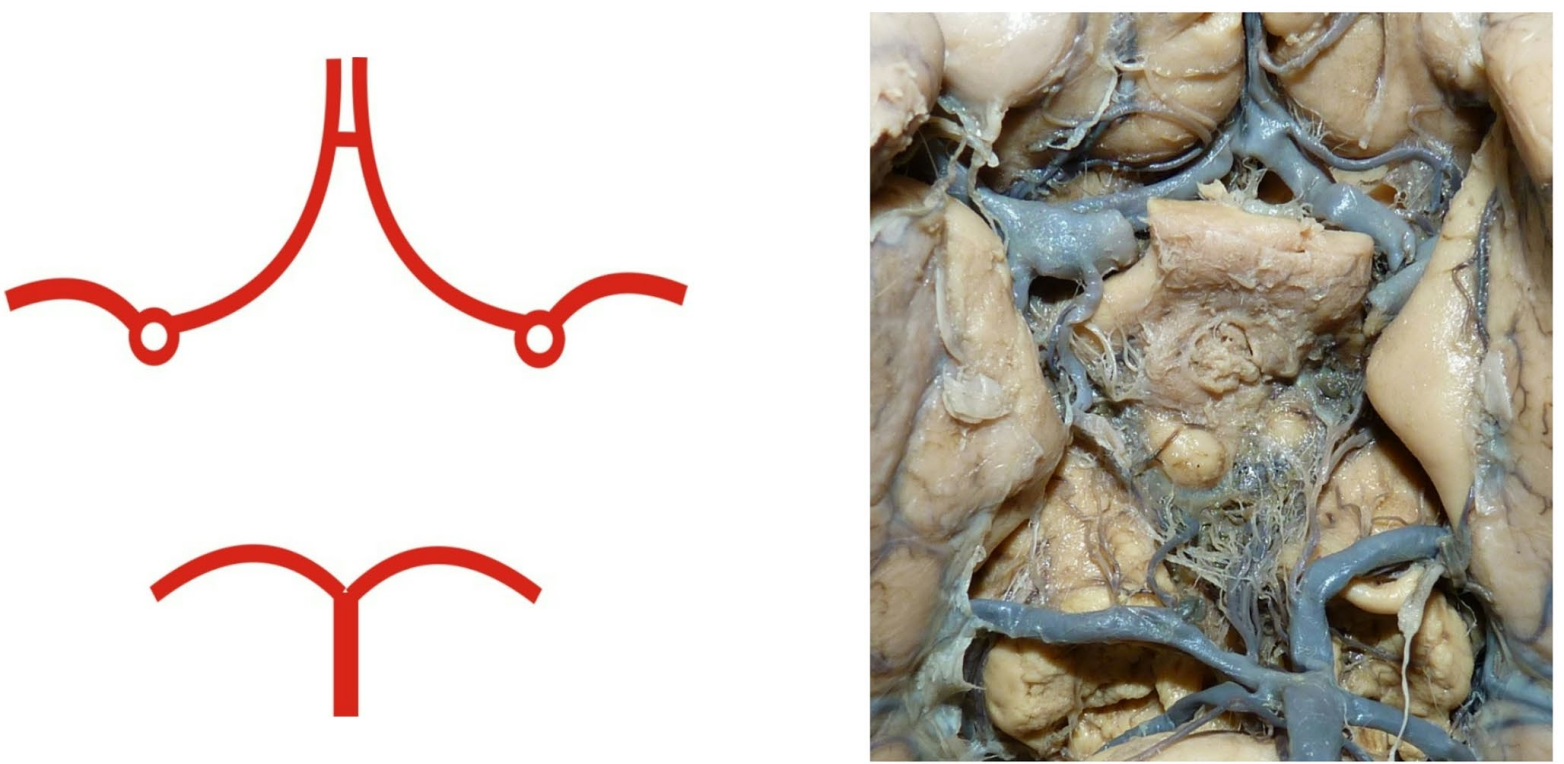
Variant g. (**A**) Schematic representation. (**B**) Representative anatomic specimen

This configuration corresponds to bilateral anatomical absence or imaging non-visualization of the posterior communicating arteries and markedly reduces the capacity for anterior-to-posterior collateral recruitment. In the present series, this pattern was identified in 11 cases (1.6%) (Fig. 9).

### Variant h

Bilateral absence of the P1 segments with basilar hypoplasia.

**Fig. 10:**
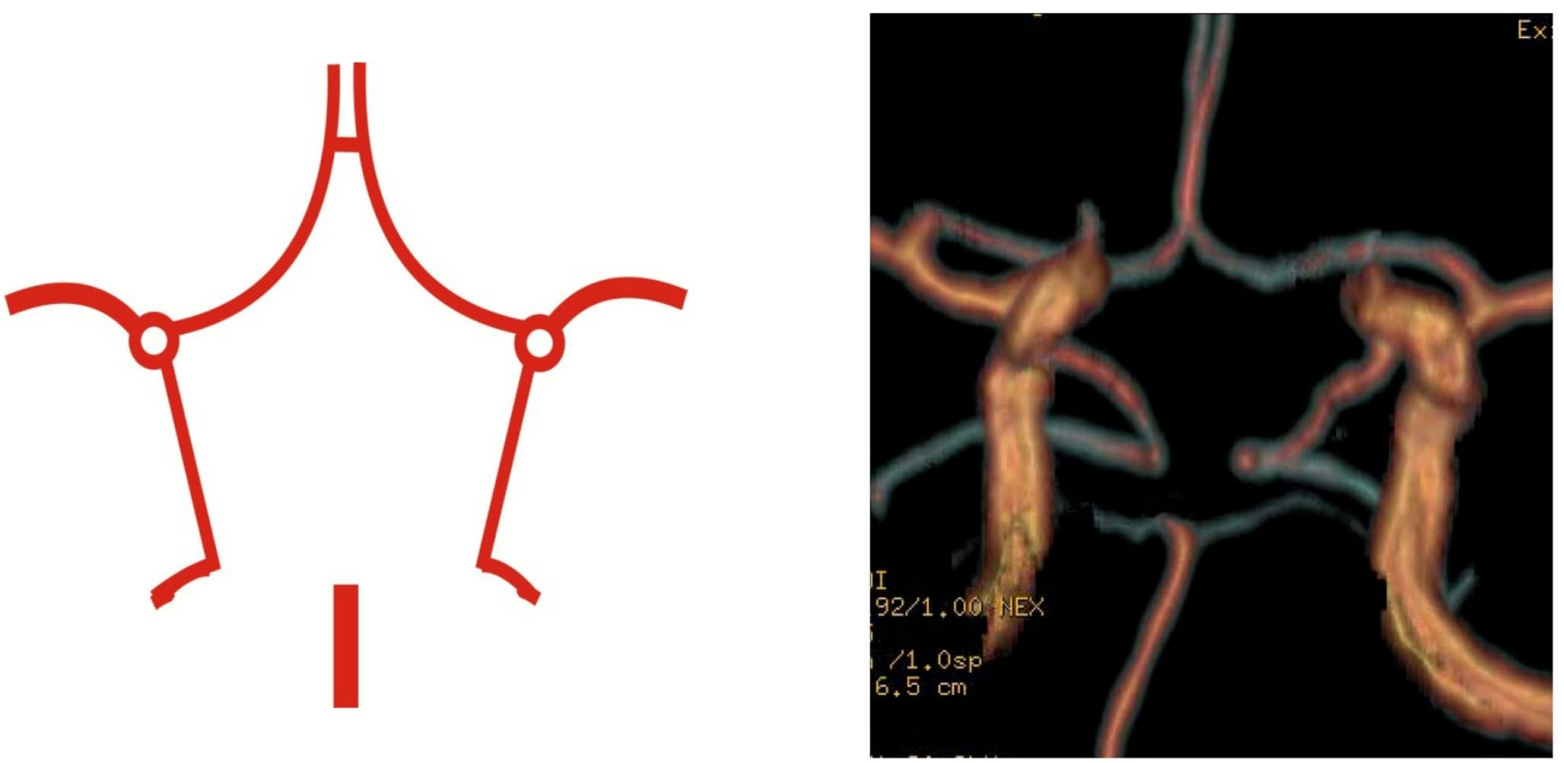
Variant h. (**A**) Schematic representation. (**B**) Representative volume-rendered CTA

This configuration corresponds to bilateral anatomical absence or imaging non-visualization of the P1 segments with associated basilar hypoplasia, resulting in bilateral carotid-dependent supply of the posterior cerebral territories and profoundly altered posterior collateral architecture. In the present series, this pattern was identified in 3 cases (0.4%) (Fig. 10).

### Variant i

Absence of one P1 segment associated with absence of the contralateral posterior communicating artery.

**Fig. 11:**
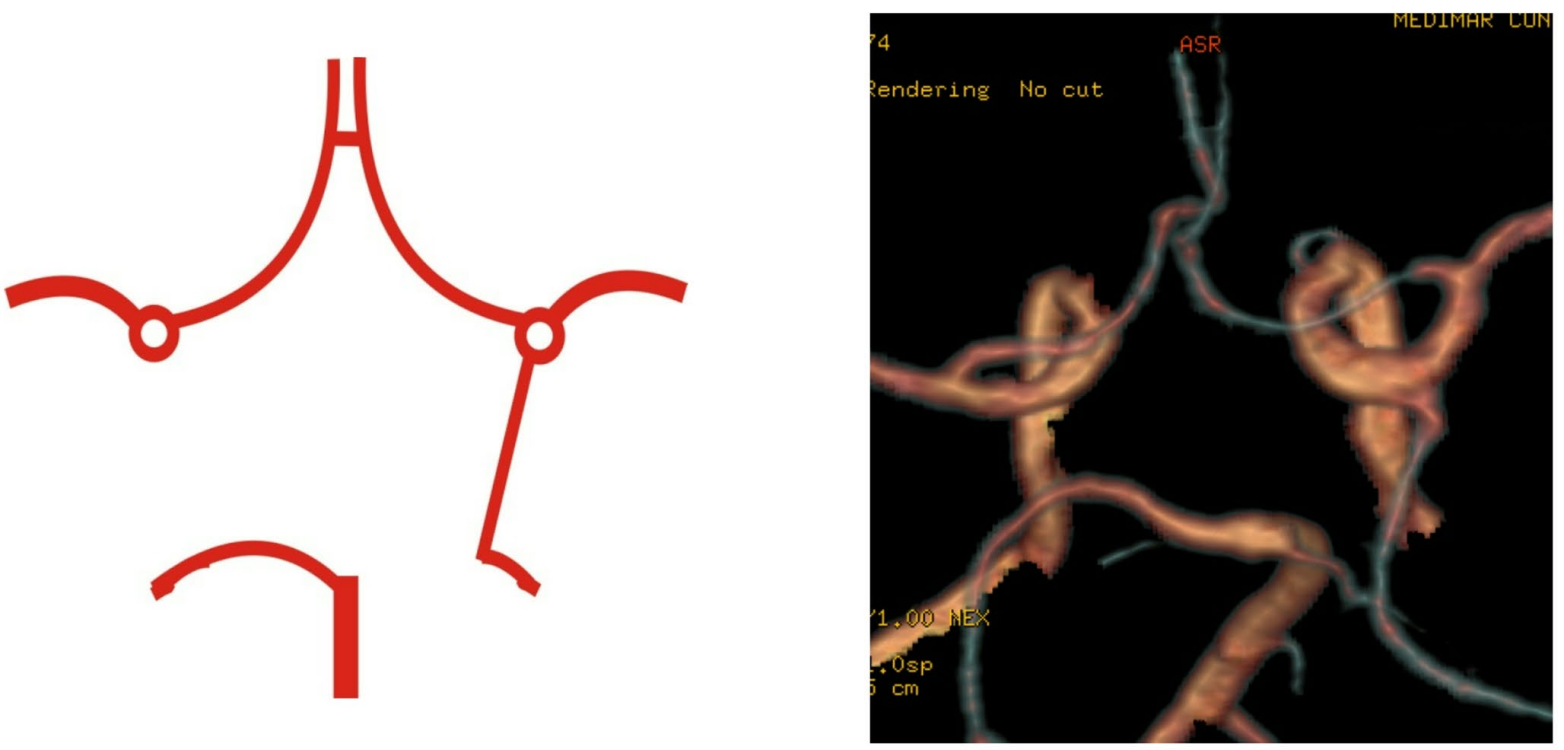
Variant i. (**A**) Schematic representation. (**B**) Representative volume-rendered CTA

This mixed asymmetrical variant combines anatomical absence or imaging non-visualization of one P1 segment with anatomical absence or imaging non-visualization of the contralateral posterior communicating artery. It leaves neither side with a complete conventional carotid-posterior connection and therefore produces a highly unbalanced posterior collateral arrangement. In the present series, this pattern was identified in 5 cases (0.7%) (Fig. 11).

### Variant j1

Incomplete posterior anastomosis due to P1 hypoplasia.

**Fig. 12:**
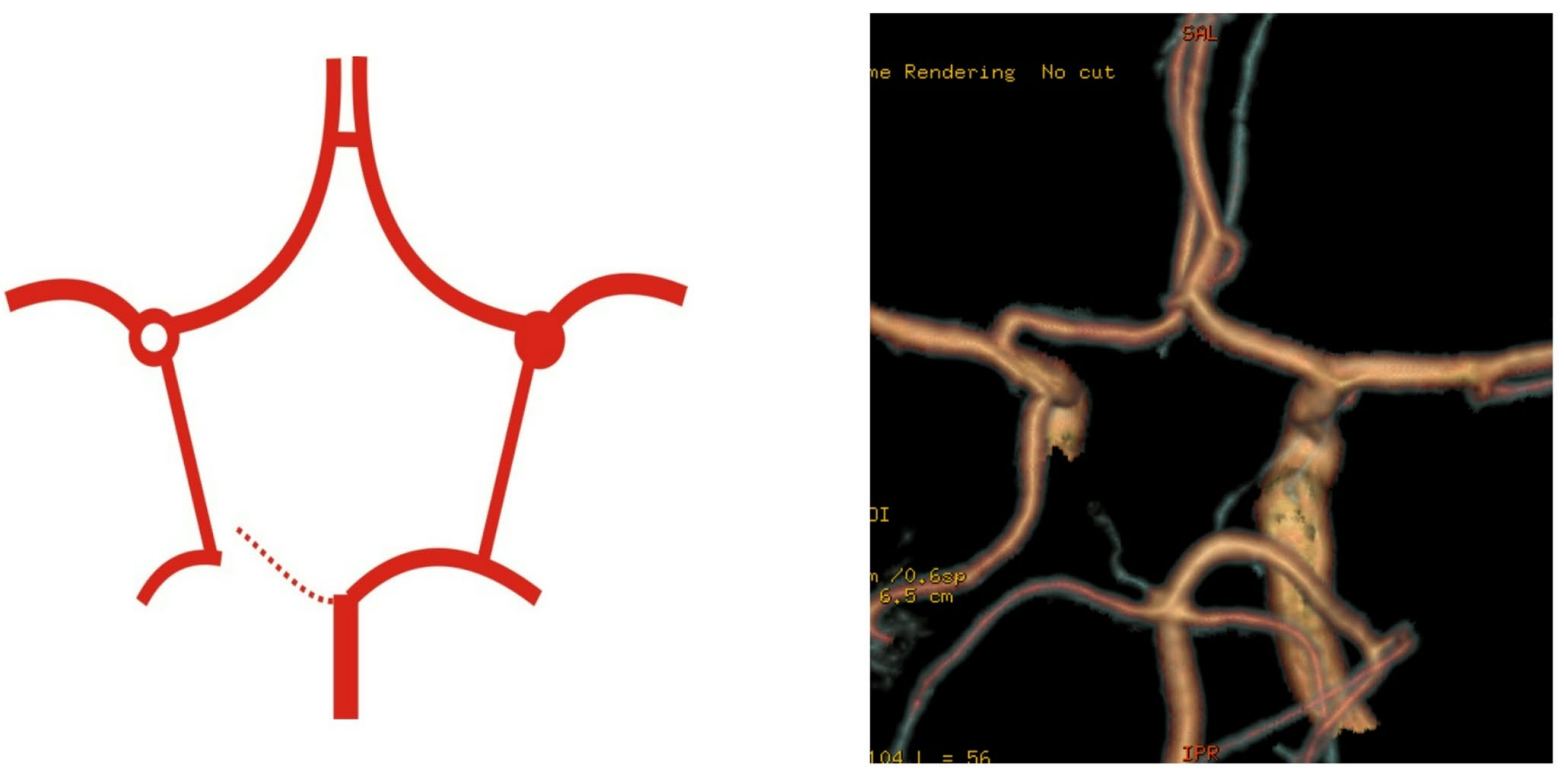
Variant j1. (**A**) Schematic representation. (**B**) Representative volume-rendered CTA

Although the posterior communicating artery and posterior cerebral artery are anatomically adjacent, the posterior anastomosis remains functionally incomplete because the P1 segment is too hypoplastic to sustain an effective communication. In the present series, this pattern was identified in 7 cases (1.1%) (Fig. 12).

### Variant j2

Incomplete posterior anastomosis due to posterior communicating artery hypoplasia.

**Fig. 13:**
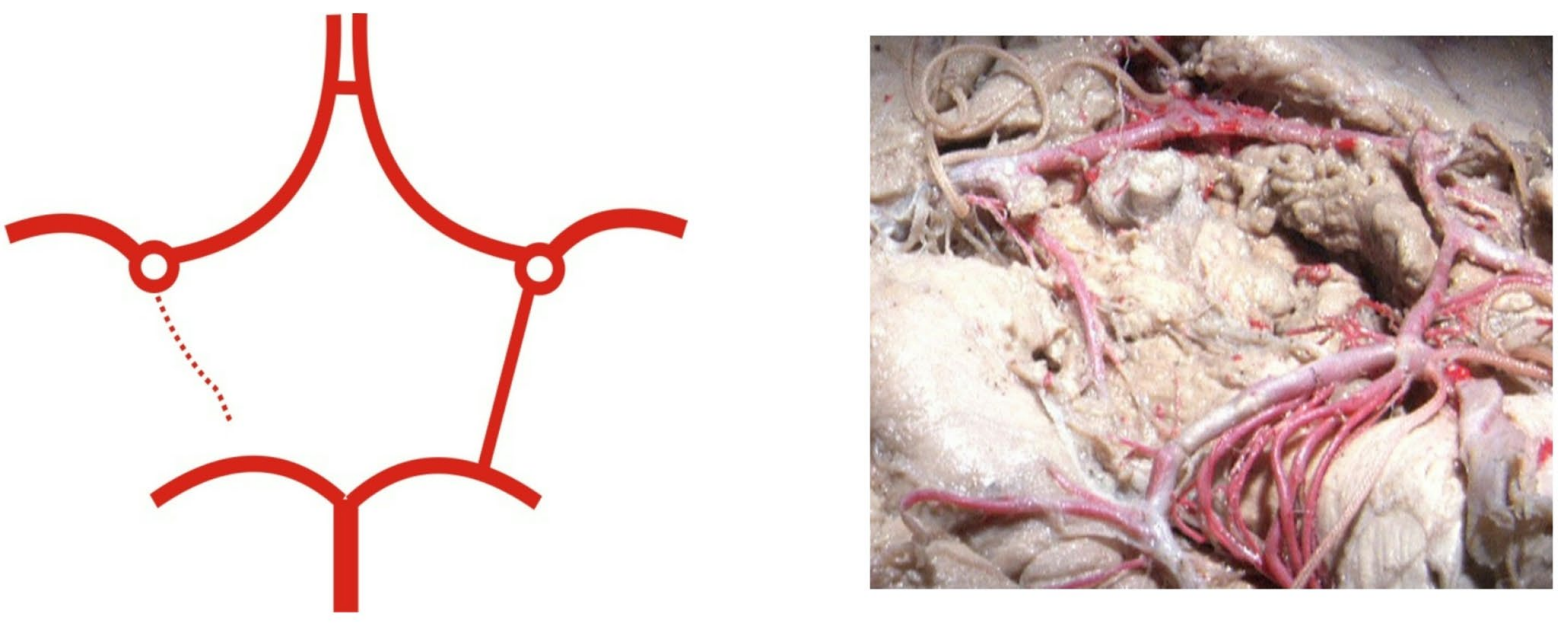
Variant j2. (**A**) Schematic representation. (**B**) Representative anatomic specimen

In this subtype, the lack of effective posterior communication is related instead to marked hypoplasia of the posterior communicating artery. The variant is especially relevant on MRA, where a very small communicating segment may be visible yet functionally insufficient. In the present series, this pattern was identified in 9 cases (1.4%) (Fig. 13).

## Imaging and clinical relevance

From a neuroradiologic perspective, atypical variants of the cerebral arterial circle are relevant not only as morphologic findings, but also as modifiers of collateral architecture and vascular dependence. On CTA and MRA, these variants may alter the expected appearance of the anterior communicating complex, reduce or abolish carotid-posterior continuity, or simulate complete configurations that are functionally incomplete [3, 4, 6, 8, 11, 15, 17].

A first practical issue is the distinction among aplasia, hypoplasia, and flow-related non-visualization. This is particularly important for the posterior communicating arteries and P1 segments, which are often small and may be incompletely depicted on routine noninvasive studies. TOF-MRA is flow dependent and may underrepresent slow-flowing or very small vessels, whereas CTA depicts the lumen more directly but may still be limited in borderline cases [6, 8, 15, 17].

In ischemic cerebrovascular disease, the relevance of these variants lies in their effect on proximal collateral capacity. Variants that interrupt the anterior communicating pathway or diminish carotid-posterior continuity may reduce the ability of the cerebral circulation to redistribute flow rapidly after carotid or vertebrobasilar compromise [11, 17].

The same anatomic patterns are also relevant to aneurysm pathophysiology. Asymmetric inflow conditions at the anterior and posterior communicating regions may contribute to aneurysm-prone hemodynamic environments, particularly in the presence of absent or hypoplastic A1, PComA, or P1 segments [3].

## Discussion

The main finding of the present study is that a substantial proportion of cerebral arterial circles cannot be adequately captured by the 22-type classification proposed by Guy Lazorthes. Although the Lazorthes framework remains historically and morphofunctionally valuable, the present material demonstrates that altered polygonal architecture, embryonic substitutions, and incomplete anastomotic arrangements extend beyond its original typologic boundaries.

Most atypical configurations in the present series involved the posterior part of the arterial circle. This predominance is consistent with modern population-based imaging studies showing that the posterior communicating arteries and P1 segments are among the most variable components of the Circle of Willis [4, 5, 8]. The present data therefore support the view that posterior collateral architecture is intrinsically less stable than the anterior communicating configuration.

The distribution of atypical variants observed in the present series should also be interpreted in the broader context of population-based and cadaveric studies of Circle of Willis morphology. Eftekhar et al. emphasized that the reported frequencies of Circle of Willis variants may differ across populations and are also influenced by the methodology used to define hypoplasia, aplasia, and completeness of the arterial circle [2]. Similarly, Nyasa et al. confirmed in a cadaveric African series that posterior segment variability is particularly frequent and that comparison across studies requires caution because of differences in classification criteria and study design [9]. A literature review and meta-analysis by Jones et al. further highlighted the marked variability of the posterior communicating artery, reinforcing the relevance of posterior atypical patterns such as those identified in the present study [5]. Finally, the systematic review by Ayre et al. demonstrated the need for standardized classification systems for Circle of Willis anatomy, which supports our effort to characterize variants that fall outside the classical Lazorthes typology using a contemporary morphologic and neuroradiologic framework [1].

Recent reports further support the relevance of detailed analysis of the anterior cerebral-anterior communicating complex in the interpretation of atypical arterial configurations. Saha et al. emphasized the considerable morphologic and morphometric variability of the anterior cerebral-anterior communicating artery complex, highlighting the need for careful structural assessment of this region when classifying anterior variants of the arterial circle of the brain [13]. In addition, Uchino and Andoh described a rare bilateral arterial ring at the anterior cerebral artery-anterior communicating artery junction associated with unilateral A1 aplasia, diagnosed by magnetic resonance angiography, which is particularly relevant to the present study because it reinforces the existence of uncommon anterior configurations that may not be fully accommodated by classical classification systems [16].

The variants classified here as j1 and j2 are particularly informative because they emphasize the distinction between anatomic continuity and effective collateral function. A circle that appears nearly complete may remain physiologically weak if the connecting segment is too hypoplastic to sustain meaningful cross-flow. This interpretation is consistent with contemporary neuroradiologic and stroke literature, which increasingly privileges collateral competence over idealized geometric completeness [11, 15, 17]. Persistent trigeminal artery variants, although rare, further illustrate why a modern interpretation of cerebral arterial anatomy must integrate morphology, embryology, and imaging. In these cases, the conventional posterior communicating pathway is absent, yet carotid-basilar communication is preserved through an embryonic channel [10, 14].

## Limitations of the study

Several limitations of the present study should be acknowledged. First, the study had a retrospective descriptive design and combined material obtained from different modalities, including dissection, injected specimens, DSA, CTA, and MRA. Although this multimodal approach increased the breadth of morphologic evaluation, it also introduced heterogeneity in the level of anatomical detail available from each method. Second, direct morphologic absence and imaging non-visualization cannot be considered fully equivalent, particularly for small posterior communicating arteries and P1 segments, where severe hypoplasia or flow-related non-visualization may mimic aplasia. Third, the study was descriptive and did not include hemodynamic modeling, perfusion analysis, or direct clinical outcome correlation. Accordingly, the neuroradiologic and collateral implications discussed in this manuscript should be interpreted as anatomically and physiologically plausible rather than directly outcome proven.

The multimodal design of the study is nevertheless a strength. By combining dissection, plastic injection, DSA, CTA, and MRA acquired in two complementary institutional settings, the series captures both direct morphologic anatomy and in vivo vascular imaging. Taken together, these findings support the interpretation of atypical variants not as marginal anomalies, but as configurations of altered collateral architecture that may be relevant to stroke imaging, aneurysm-related hemodynamics, and neurovascular planning.

## Conclusions

The anatomy of the cerebral arterial circle cannot be fully encompassed by the 22 variants described by Guy Lazorthes. In the present series, a relevant subset of cases displayed configurations outside this classification, including altered polygonal architecture, anatomical absence or imaging non-visualization of one or more segments, incomplete posterior anastomosis, and persistence of embryonic carotid-basilar channels.

These atypical variants may influence collateral capacity, territorial dependence, and the interpretation of CTA and MRA. They should therefore be regarded as clinically relevant patterns of altered collateral architecture.

## Data Availability

No datasets were generated or analysed during the current study.

## References

[1] Ayre JR, Bazira PJ, Abumattar M, Makwana HN, Sanders KA (2022) A new classification system for the anatomical variations of the human circle of Willis: a systematic review. J Anat 240:1187–1204. 10.1111/joa.13636 34936097 10.1111/joa.13616 PMC9119622

[2] Eftekhar B, Dadmehr M, Ansari S, Ghodsi M, Nazparvar B, Ketabchi E (2006) Are the distributions of variations of circle of Willis different in different populations? Results of an anatomical study and review of literature. BMC Neurol 6:22. 10.1186/1471-2377-6-22 16796761 10.1186/1471-2377-6-22 PMC1543654

[3] Feng L, Mao HJ, Zhang DD, Zhu YC, Han F (2023) Anatomical variations in the Circle of Willis and the formation and rupture of intracranial aneurysms: a systematic review and meta-analysis. Front Neurol 13:1098950. 10.3389/fneur.2022.1098950 36726753 10.3389/fneur.2022.1098950 PMC9885143

[4] Hindenes LB, Haberg AK, Johnsen LH, Mathiesen EB, Robben D, Vangberg TR (2020) Variations in the Circle of Willis in a large population sample using 3D TOF angiography: the tromso study. PLoS ONE 15:e0241373. 10.1371/journal.pone.0241373 33141840 10.1371/journal.pone.0241373 PMC7608873

[5] Jones JD, Castanho P, Bazira P, Sanders K (2021) Anatomical variations of the circle of Willis and their prevalence, with a focus on the posterior communicating artery: a literature review and meta-analysis. Clin Anat 34:978–990. 10.1002/ca.23662 32713011 10.1002/ca.23662

[6] Karatas A, Coban G, Cinar C, Oran I, Uz A (2015) Assessment of the circle of Willis with cranial tomography angiography. Med Sci Monit 21:2647–2652. 10.12659/MSM.894322 26343887 10.12659/MSM.894322 PMC4576924

[7] Lazorthes G, Gouaze A, Santini JJ, Salamon G (1979) The arterial circle of the brain (circulus arteriosus cerebri). Anat Clin 1:241–257. 10.1007/BF01641237

[8] Li J, Liu X, Xu W et al (2020) Examination of structural variations of the Circle of Willis by 3D time-of-flight magnetic resonance angiography. Med (Baltim) 99:e19290. 10.1097/MD.0000000000019290 10.3389/fnins.2020.00071 PMC7026468 32116517

[9] Nyasa C, Mwakikunga A, Tembo L, Dzamalala C, Ihunwo AO (2021) Distribution of variations in anatomy of the circle of Willis: results of a cadaveric study of the Malawian population and review of literature. Pan Afr Med J 38:11. 10.11604/pamj.2021.38.11.27126 34567338 10.11604/pamj.2021.38.11.27126 PMC8444123

[10] Padget DH (1948) The development of the cranial arteries in the human embryo. Contrib Embryol 32:207–261

[11] Regenhardt RW, Das AS, Stapleton CJ et al (2023) Collateral circulation in ischemic stroke: an updated review. Brain Circ 9:113–127. 10.4103/bc.bc_28_23

[12] Riggs HE, Rupp C (1963) Variation in form of Circle of Willis. The relation of the variations to collateral circulation: anatomic analysis. Arch Neurol 8:8–14. 10.1001/archneur.1963.00460070034003 13973856 10.1001/archneur.1963.00460010024002

[13] Saha A, Bhattacharya A, Ghosh SP, Roy S (2024) Morphology and morphometry of the anterior cerebral-anterior communicating artery complex. Surg Radiol Anat 46:1585–1593. 10.1007/s00276-024-03451-5 39103573 10.1007/s00276-024-03451-5

[14] Saltzman GF (1959) Patent primitive trigeminal artery studied by cerebral angiography. Acta Radiol (Stockh) 51:329–336. 10.3109/00016925909172554 13649384 10.3109/00016925909171103

[15] Sui B, Sun YV, Matouk CC et al (2024) Report from the society of magnetic resonance angiography: clinical applications of 7T neurovascular MR in the assessment of intracranial vascular disease. J Magn Reson Imaging 59:744–758. 10.1002/jmri.28921

[16] Uchino A, Andoh S (2025) Bilateral anterior cerebral artery-anterior communicating artery junction arterial ring (duplicate origin of the A2) associated with unilateral A1 aplasia diagnosed by magnetic resonance angiography. Surg Radiol Anat 47:192. 10.1007/s00276-025-03708-7 40884554 10.1007/s00276-025-03708-7

[17] Zhang K, Xie Y, Li H et al (2024) Estimating flow direction of Circle of Willis using dynamic arterial spin-labeling magnetic resonance angiography. AJNR Am J Neuroradiol 45:1419–1426. 10.3174/ajnr.A8430 38789121 10.3174/ajnr.A8355 PMC11448990

